# Type 2 Diabetes and Alzheimer’s Disease: The Emerging Role of Cellular Lipotoxicity

**DOI:** 10.3390/biom13010183

**Published:** 2023-01-16

**Authors:** Nicola Marrano, Giuseppina Biondi, Anna Borrelli, Martina Rella, Tommaso Zambetta, Ludovico Di Gioia, Mariangela Caporusso, Giancarlo Logroscino, Sebastio Perrini, Francesco Giorgino, Annalisa Natalicchio

**Affiliations:** 1 Department of Precision and Regenerative Medicine and Ionian Area, Section of Internal Medicine, Endocrinology, Andrology and Metabolic Diseases, University of Bari Aldo Moro, 70124 Bari, Italy; 2 Department of Translational Biomedicine and Neuroscience, University of Bari Aldo Moro, 70124 Bari, Italy; 3 Center for Neurodegenerative Diseases and the Aging Brain, University of Bari Aldo Moro at Pia Fondazione Cardinale G. Panico, 73039 Lecce, Italy

**Keywords:** type 2 diabetes, Alzheimer’s disease, lipotoxicity, obesity

## Abstract

Type 2 diabetes (T2D) and Alzheimer’s diseases (AD) represent major health issues that have reached alarming levels in the last decades. Although growing evidence demonstrates that AD is a significant comorbidity of T2D, and there is a ~1.4–2-fold increase in the risk of developing AD among T2D patients, the involvement of possible common triggers in the pathogenesis of these two diseases remains largely unknown. Of note, recent mechanistic insights suggest that lipotoxicity could represent the missing ring in the pathogenetic mechanisms linking T2D to AD. Indeed, obesity, which represents the main cause of lipotoxicity, has been recognized as a major risk factor for both pathological conditions. Lipotoxicity can lead to inflammation, insulin resistance, oxidative stress, ceramide and amyloid accumulation, endoplasmic reticulum stress, ferroptosis, and autophagy, which are shared biological events in the pathogenesis of T2D and AD. In the current review, we try to provide a critical and comprehensive view of the common molecular pathways activated by lipotoxicity in T2D and AD, attempting to summarize how these mechanisms can drive future research and open the way to new therapeutic perspectives.

## 1. Introduction

Diabetes is a major health issue that has reached alarming levels. According to the International Diabetes Federation, today, approximately 537 million people (aged 20–79) are living with diabetes worldwide (more than one in 10 adults) [1]. This number is predicted to rise to 643 million by 2030 and 783 million by 2045. Diabetes was responsible for 6.7 million deaths in 2021 and caused at least USD 966 billion in health expenditure (a 316% increase over the last 15 years) [1]. Type 2 diabetes (T2D) is the most common type of diabetes, accounting for over 90% of all diabetes worldwide [1].

The causes of T2D are not entirely understood, but a strong link with obesity has long been recognised [1]. The World Health Organization defines obesity as an excessive fat accumulation that presents a risk to health [2]. Indeed, when the dietary fat surfeit exceeds the storage ability of the adipose tissue, it accumulates in ectopic sites (i.e., heart, skeletal muscle, liver, and pancreas), thus contributing to enlarging visceral deposits and resulting in free fatty acids (FFAs)-induced toxicity, also known as lipotoxicity [3]. Specifically, lipotoxicity is associated with a reduction in pancreatic beta-cell functional mass [3,4] and whole-body insulin sensitivity [5], as well as an increased risk of cardiovascular and renal disturbances [6,7,8]. These features may represent the mechanistic link between obesity, T2D, and its complications.

Alzheimer’s disease (AD) has long been recognized as a critical comorbidity of T2D [9]. AD is a neurodegenerative disease involving cognitive impairment, neuronal dysfunction, and memory loss, and it is considered the most common cause of dementia. Currently, 40–50 million people live with dementia, of which 26 million have AD [10,11]. Notably, numerous studies [12,13,14,15,16], including recent meta-analyses [17,18], confirmed that there is a ~1.4–2-fold increase in the risk of developing AD among T2D patients.

It is well known that impaired glucose metabolism, insulin resistance, inflammation, oxidative stress, amyloid accumulation, advanced glycosylation end products accrual, and mitochondrial dysfunction are biological events that occur in both T2D and AD (reviewed in [9,19]), such that AD has been recently designated as “type 3 diabetes” [20] or “diabetes of the brain” [21]. Nevertheless, the existence of common molecular mechanisms underlying these events and the involvement of possible common triggers remain largely unknown. Of note, as for T2D, obesity has been recognized as a significant risk factor also for cognitive decline and AD [22,23,24,25]. More specifically, recent mechanistic insights provided by animal models and in vitro experiments suggest that lipotoxicity could contribute to neurological dysfunction and neurodegeneration [26,27,28], possibly representing the missing ring in the pathogenetic mechanisms linking T2D to AD.

In the current review, we try to provide a critical and comprehensive view of the common molecular pathways activated by lipotoxicity in T2D and AD, attempting to summarize how these mechanisms can drive future research and open the way to new therapeutic perspectives.

## 2. Lipotoxicity Common Mechanism in Type 2 Diabetes and Alzheimer’s Disease

### 2.1. Inflammation

Low-grade chronic systemic inflammation is an established hallmark of lipotoxicity [29,30,31]. During obesity, the expanded adipose tissue secretes proinflammatory factors and the FFAs, which reach the bloodstream and lead to tonic activation of the innate immune system and to maladaptive responses, such as fibrosis, necrosis, and altered secretion of local proinflammatory factors, which cause significant tissue damage in multiple organs, including pancreas, liver, skeletal muscle, heart, and brain [32,33,34,35,36]. Different lipid species that are elevated due to diet or obesity may also directly contribute to tissue inflammation, triggering the proinflammatory cascade initiated by toll-like receptors (TLRs), such as TLR4, and altering the release of chemokines and cytokines by several tissues [37].

Much evidence supports an association between lipotoxicity-induced inflammation and the pathogenesis and progression of T2D. Indeed, hormones, cytokines, and fatty acids secreted by the inflamed adipose tissue can drive beta-cell failure in the transition from obesity to diabetes [3]. For example, saturated FAs (SFAs), whose concentrations are increased in obesity or following a high-fat diet (HFD), can induce beta-cell production of interleukin 1 (IL-1) [38,39,40], a cytokine able to increase the local expression of proinflammatory molecules, leading to local inflammation, apoptosis, and impaired insulin secretion [41].

In insulin-sensitive tissues (e.g., adipose tissue, liver, and muscle), FFAs can promote tissue inflammation by binding TLR2 and -4, resulting in nuclear factor (NF)-κB and c-Jun N-terminal kinase (JNK) activation. Once activated, these pathways can increase the local synthesis and secretion of proinflammatory cytokines and chemokines, leading to the infiltration of proinflammatory macrophages, impairment of intracellular signaling, and disruptions of metabolic physiology [42].

As for T2D, much evidence suggests that inflammation induced by lipotoxicity (in particular, the local neuroinflammation) is a key mechanism contributing to the pathogenesis and progression of AD [43,44,45,46], and markers of neuroinflammation have been observed in the brain of AD models [47]. Of note, multiple lines of evidence highlight the link between lipotoxicity-induced peripheral inflammation and neuroinflammation [48,49]. Indeed, during obesity, adipose tissue-derived proinflammatory cytokines [3] can pass through the blood-brain barrier (BBB) and induce local cytokine production, disrupting neural circuits involved in cognition and memory [50,51]. Studies on murine models have shown that HFD feeding is associated with astrocyte deformation, microglial activation, and higher levels of IL-1β, IL-6, tumor necrosis factor-α (TNF-α), and interferon-γ in the hippocampus, which directly impaired cognitive function, including learning and memory activities [52]. In animal models, diet-induced obesity increases inflammatory responses also in other brain regions, such as the cerebral cortex [53] and hypothalamus [54]. Central inflammation is also likely to be exacerbated by lipotoxicity-induced damage to the BBB [25,55,56]. Indeed, high circulating levels of SFAs can induce the expression of inflammatory mediators and decrease the expression of tight junction proteins in brain microvascular endothelial cells, thus compromising the integrity of the BBB. This event causes the entry of inflammatory signals, FFAs, and immune cells into the brain, and the consequent activation of microglia [57], which, in turn, can act on vascular endothelial cells, leading to a vicious cycle [58].

Regardless of the induction of peripheral inflammation and the effects on BBB, dietary FAs can directly influence the inflammatory phenotype in the central nervous system [59]: SFAs and mono-unsaturated FAs affect the NF-κB pathway, activate TLR-4 receptors, induce proinflammatory cytokines (IL-1β, IL-6, and TNF-α) [60,61], and increase oxidative stress and endoplasmic reticulum (ER) stress, which are all risk factors for AD [62,63,64,65], leading to excessive glial and microglia activation and to pathological phosphorylation of tau, a microtubule-associated protein involved in the assembly of neurofibrillary tangles, all of which are hallmarks of AD [66]. It has been demonstrated that conditioned media from palmitate-stimulated astrocytes induce AD-like hyperphosphorylation of tau in primary rat cortical neurons [67,68]. Of note, in primary astrocytes, palmitate activates the IL-1β-releasing NLR family CARD domain containing 4 (NLRC4) inflammasome, whose levels are upregulated in the brains of AD patients, thus suggesting a possible role also of the NLRC4 inflammasome in AD pathogenesis [69].

In conclusion, lipotoxicity is associated with both peripheral and neural inflammation, which represents a key mechanism contributing to the onset and progression of T2D and AD [70].

### 2.2. Insulin Resistance

Insulin resistance is a condition characterized by a relative inability of target tissues to respond to insulin action due to the downregulation of insulin receptor (IR) expression, its inability to bind insulin, or faulty activation of the insulin signalling cascade [71]. When accompanied by the dysfunction of pancreatic beta-cells, insulin resistance results in the failure to control blood glucose levels, thus representing a hallmark in the pathogenesis of T2D [72]. Obesity has long been recognized as the primary trigger of insulin resistance: during obesity, the increased release of FFAs, hormones, and proinflammatory cytokines by the adipose tissue promotes the induction of peripheral insulin resistance through several molecular mechanisms, among which inflammation and oxidative stress [73,74]. In peripheral insulin-sensitive tissues, high levels of FFAs can result in an increased intracellular content of FAs metabolites, such as diacylglycerol (DAG), fatty acyl-coenzyme A (fatty acyl-CoA), and ceramides, which, in turn, inhibit insulin signaling [75]. For instance, in hepatocytes, DAG activates protein kinase C ε (PKCε), which phosphorylates and inhibits the IR, resulting in reduced insulin receptor substrate 2 (IRS-2) tyrosine phosphorylation and in the inability of insulin to activate hepatic glycogen synthesis and suppress hepatic glucose production [76]. Likewise, the accumulation of intracellular FAs in muscular tissue positively correlates with the decrease in muscular tissue sensitivity to insulin since DAG and ceramides can activate PKC, which is able to phosphorylate IRS-1 on serine residues, thus impairing the activation of PI3K and insulin signalling [77]. It should be noted that lipotoxicity-induced insulin resistance, typically recognized in insulin-sensitive tissues, has recently been demonstrated also in pancreatic beta-cells, thus representing a new mechanism of lipotoxic damage contributing to the reduction of beta-cell functional mass and the pathogenesis of T2D [78].

As described for T2D, insulin resistance, mainly caused by lipotoxicity, is known to be actively involved in the pathophysiology of AD. In mouse models of AD, an HFD-inducing peripheral and central insulin resistance increases amyloidosis in the brains [79,80]. Other human studies have suggested the presence of brain insulin resistance in obesity [81,82]. Accordingly, it has been shown that melatonin alleviates cognitive impairments by reducing brain insulin resistance in elderly rats on a HFD [83] and that intranasal insulin administration improves memory and other cognitive functions in healthy adults with obesity [84]. Under lipotoxic conditions, FFAs can cross the BBB, infiltrate into the cerebrospinal fluid, in concentration depending on plasma fatty acid levels [27,85], and accumulate in the brain as long chain-acyl CoA esters, DAG, and ceramides, causing central lipotoxicity and insulin resistance, as observed in peripheral insulin-sensitive tissues [86,87]. In particular, it has been demonstrated that the hypothalamic de novo ceramide synthesis plays a crucial role in central insulin resistance development and glucose homeostasis dysregulation associated with obesity [87]. In addition, also in the brain, impaired insulin signaling induced by lipotoxicity can also be associated with dysfunctional mitochondria and increased reactive oxygen species (ROS) production [88].

### 2.3. Oxidative Stress

Oxidative stress is defined as an imbalance in antioxidants and pro-oxidants in favour of the latter, associated with a transient or long-term increase in ROS and reactive nitrogen species (RNS) levels, disruption of redox circuits, alteration of metabolic and signaling pathways, and macromolecular damage, which culminate in cellular dysfunction and death [89,90]. Prolonged overnutrition (particularly the excess of FAs) leads to chronic ROS and RNS production, which promotes oxidative stress in cells, tissues, and organs. Lipotoxicity-induced oxidative stress results in damage to cell membranes, DNA, and proteins, as well as modulation of the activity of transcriptional factors through redox chemistry, including NF-𝜅B, leading to chronic inflammation, insulin resistance, and cell apoptosis [91].

Because of the high content of lipids, the high oxygen consumption rate, and the scarcity of antioxidant defence mechanisms, the human brain is highly susceptible to ROS insults and oxidative stress [92], which can play a major role in the neurodegenerative process in the AD brain [93,94].

It has been demonstrated that HFD exacerbates AD-related pathology through mechanisms involving oxidative stress. Indeed, in animal models of AD, HFD-induced oxidative stress in cerebral microvasculature induces the dysfunction of pericytes which alters BBB functionality, thus leading to amyloid accumulation in the brain [95].

As mentioned above, during obesity, FFAs cross the BBB and infiltrate into the brain [56], thus activating microglial inflammatory pathways and increasing the production of proinflammatory cytokines and ROS [63,64,65,96]. Interestingly, altered intracerebral levels of specific SFAs and/or unsaturated FAs, as well as changes in their ratio, may contribute differently to the oxidative stress-mediated pathogenesis of AD [97].

Like neurons, pancreatic beta-cells are also characterised by a low expression of antioxidant enzymes [98], thus being more vulnerable to oxidative stress. Several studies demonstrate that lipotoxicity induces beta-cell oxidative stress, thus impairing beta-cell function and survival [99,100,101] and promoting the onset and progression of T2D [102]. As observed in the brain, chain length and degree of saturation of FAs are crucial factors for lipotoxicity. For instance, very long-chain FAs increased hydrogen peroxide (H_2_O_2_) generation more potently than long-chain FAs in both beta-cells peroxisomes and mitochondria [103]. We have previously demonstrated that, in pancreatic beta-cells, high levels of long-chain SFAs (i.e., palmitate) increase the expression and activation of the p66^Shc^ protein, a redox adaptor protein acting as both a sensor and a producer of ROS, thus inducing beta-cell apoptosis [104].

Oxidative stress can be considered both a cause and a consequence of lipotoxicity-induced mitochondrial dysfunction, which is an important pathological mechanism in both T2D and AD [105]. Lipotoxicity-induced mitochondria dysfunction can lead to insulin resistance [106] and cell death in several tissues involved in the pathogenesis and progression of both T2D and AD [107]. FAs can disrupt the normal proton gradient in the mitochondria intermembrane space during electron transport, increasing mitochondrial proton conductance. The subsequent dissipation of the proton gradient inhibits the oxidative phosphorylation of ADP by ATP synthetase, ultimately leading to the release of Ca^2+^, energy crisis, and cell demise [108,109]. Moreover, FFAs can cause an exaggerated activation of the inducible form of nitric oxide synthase (iNOS), resulting in the generation of excessive nitric oxide (NO), subsequent mitochondrial DNA damage, and beta-cell apoptosis [110].

Increased levels of heme-oxygenase-1 (HO-1) protein could represent another oxidative stress mechanism induced by lipotoxicity, common to the pathogenesis of T2D and AD [111,112]. Indeed, the activation of HO-1 results in high levels of carbon monoxide (CO) and ferrous iron (Fe^2+^), which impact insulin secretion in beta-cells [113]. Similarly, CO and Fe^2+^ can modulate hippocampal synaptic activity, being neurotoxic at high levels [114]. Moreover, increased levels of HO-1 have been correlated with both insulin resistance [115] and increased levels of brain oxidative markers [113].

Finally, the nicotinamide adenine dinucleotide phosphate (NADPH) oxidases (NOXs) proteins could be a further possible common mediator of lipotoxicity-induced oxidative stress in T2D and AD. Indeed, SFAs increase their expression and activation [116], leading to beta-cell dysfunction and apoptosis [117,118,119]. Inappropriate activation of NOXs enzymes may also damage the liver, adipose tissue, and other organs, playing an important role in obesity-induced metabolic syndrome and diabetes [120,121,122]. Interestingly, lipotoxicity also stimulates NOXs activation in micro- and macro-vascular tissues of animal models and patients with diabetes and obesity, thus playing an important role in diabetic vascular complications. On the other hand, it has been demonstrated that, in diet-induced obesity, NOX-mediated oxidative stress induces cerebral vascular dysfunction [123]. In addition, long-term HFD leads to NOXs activation, disturbed brain insulin signaling, and tau protein hyperphosphorylation/aggregation in rats [124].

### 2.4. Ceramides

Under lipotoxic conditions, the FFAs accumulated in non-adipose tissues are metabolized through the de novo synthesis pathway into lipid derivatives, such as ceramides and other sphingolipids (e.g., sphingomyelin and sphingosine) [125]. Although physiological ceramide levels exert important biological effects, by altering the physical-chemical properties of the lipid bilayers and regulating the activity of intracellular receptors and proteins [126], their excessive accumulation triggers a series of cellular stress responses, leading to apoptosis in different tissues and contributing to the pathogenesis of diseases such as T2D and AD [127,128,129].

Pancreatic beta-cell lipotoxicity induced by ceramide accumulation was first reported in 1998 when Shimabukuro et al. [130] demonstrated that beta-cell apoptosis was mediated by FAs-enhanced ceramide synthesis in a rat model of obesity and T2D. Increased ceramide content and pancreatic islet apoptosis were associated with increased expression of serine palmitoyl transferase (SPT), a key enzyme in the de novo synthesis of ceramides, while apoptosis was prevented by the SPT inhibitor fumonisin B1 [130]. Similarly, the prolonged exposure of human pancreatic islets to a mixture of FFAs led to an increase in apoptosis which was partially reduced by a ceramide synthesis inhibitor [131]. Ceramides can induce apoptosis in pancreatic beta-cells through several pathways, including Akt inhibition, activation of JNK and extracellular signal-regulated kinases (ERK), dephosphorylation of B-cell lymphoma 2 (Bcl-2), and activation of the Bcl-2-associated death promoter (Bad). Ceramides can directly activate cathepsin D, which is responsible for activating of the proapoptotic proteins Bid and Bax, thus resulting in lipoapoptosis [108]. Furthermore, chronic exposure of mouse beta-cells to palmitate has been shown to cause an alteration of ER sphingolipids composition, including a reduction in sphingomyelin and an accumulation of ceramides, resulting in the destruction of the ER lipid raft and, therefore, in ER stress [132].

In addition to beta-cells, the accumulation of ceramides can occur in other insulin-sensitive tissues in obesity, such as adipose tissue and skeletal muscle, contributing to the development of insulin resistance and, therefore, to the pathogenesis of T2D [133,134,135].

As described for T2D, lipotoxicity-induced ceramide accumulation is a mechanism also involved in the pathogenesis of AD. Ceramide accumulation in the brain could be directly due to the increase in FFAs levels or indirectly promoted by the FFAs-induced neuroinflammation under lipotoxic conditions [136]. As mentioned above, high levels of FFAs can enter and be metabolized in the brain, resulting in increased ceramide synthesis [137]. Concordantly, an increase in ceramide levels has been observed in the brains of patients with early stages of AD [138].

Of note, elevated serum ceramide levels can also increase the risk of developing AD [139]. Indeed, peripherally synthesized ceramides, released into the bloodstream under conditions of obesity or T2D, can transit across the BBB, alter insulin signaling and induce insulin resistance in the brain [140]. Concordantly, the exposure of neuronal cells to ceramides has been shown to alter the expression of several genes critical for insulin and insulin-like growth factor (IGF) signaling pathways, including insulin, IR, IGF-1 and IGF-2 receptors, IRS-1, and IRS-4 [141]. In addition, the accumulation of ceramides can directly contribute to neuronal apoptosis through the generation of ROS and the inactivation of the PI3K/Akt signaling, leading to mitochondria-mediated apoptosis [142].

### 2.5. Amyloid Accumulation

Amyloid accumulation is a typical pathological feature of various diseases, including AD and T2D. Specifically, T2D is characterized by extracellular amyloid deposits of the islet amyloid polypeptide (IAPP) in the pancreas, while AD is marked by the accumulation of amyloid-β (Aβ) plaques in the brain.

IAPP or amylin is a peptide physiologically produced by beta-cells, packaged in secretory granules, and secreted with insulin in response to secretagogues, including glucose [143]. During obesity, insulin resistance and the associated increase in insulin secretory demand lead to the overproduction of IAPP which, when in excess, is deposited and tends to form aggregates [144].

Several studies report the ability of FFAs to directly increase IAPP mRNA levels and to promote its protein secretion and thus its aggregation [145,146]. Furthermore, in human IAPP (hIAPP) transgenic mice, dietary fat enhances both the prevalence and severity of islet amyloid deposition, leading to beta-cell loss and impaired insulin secretion [147]. Of note, hIAPP aggregates can induce ER stress, oxidative stress, and apoptosis, thus being cytotoxic for beta-cells [144,148,149,150]. The high concentration of hIAPP can also cause fibril formation in secretory granules that, once released by exocytosis, can interact with the cell membrane causing cytotoxicity or settling as fibrils in the extracellular space [151].

On the other hand, senile plaques composed of Aβ peptides and hyperphosphorylated tau proteins enriched neurofibrillary tangles are the main histopathological features of AD. Many factors can affect the accumulation and aggregation of Aβ, including dietary fat and diabetes [152]. The amyloidogenic process is believed to occur within membrane microdomains (lipid rafts) enriched in cholesterol and sphingolipids. It is, therefore, not surprising that lipotoxicity-induced alteration of lipid homeostasis in these sites can promote the formation of Aβ peptides. For instance, an increase in cholesterol levels can enhance the activity of the enzymes β- and γ-secretase that cleave the amyloid precursor protein into monomeric Aβ peptides, highly subjected to aggregation [153,154,155]. The alteration in the levels of lipids, such as ceramides, may also contribute to the pathophysiology of AD by influencing the production of Aβ. Indeed, there is evidence that ceramide analogues can modulate the activity of γ-secretases by promoting the formation of Aβ [156,157]. Of note, Aβ deposition can promote sphingomyelinase activity, thus establishing a vicious cycle that exacerbates the neurotoxicity in the brain of AD patients [158,159,160].

Interestingly, it has been observed that peripheral plasma Aβ (produced by enterocytes and associated with chylomicrons and lipoprotein B) can cross the BBB, participating in the formation of Aβ plaques in the brain [161]. In particular, increased levels of plasma Aβ protein, derived from a diet rich in FAs and cholesterol, lead to the dysfunction of the BBB and, therefore, to an exaggerated delivery of peripheral Aβ from the blood to the brain [162,163]. This evidence further strengthens the link between lipid metabolism and the deposition of amyloid plaques in the brain.

### 2.6. ER Stress

The ER is the intracellular site where proteins are folded and assembled [164]. When the demand to synthesize and process proteins exceeds the ER capacity, misfolded or unfolded proteins can accumulate in the ER and cause the so-called “ER stress”. In these cases, the unfolded protein response (UPR) is activated in the cell, which initially leads to a better folding rate and degradation of misfolded proteins. However, if this adaptive response is insufficient and ER stress persists, a series of mechanisms are activated, leading to cell dysfunction and death [164].

Beta-cells have a huge biosynthetic capability and are highly dependent on their ER, where secretory proteins, such as proinsulin, are synthesized to cope with the oscillatory requirement of secreted insulin to maintain normoglycemia. Consequently, beta-cells are extremely susceptible to ER stress, especially when overloaded. Many studies have reported a key role of FFAs, particularly SFAs, in promoting ER stress in pancreatic beta-cells. The exposure of INS-1E cells, human pancreatic islets, and MIN6 cells to palmitate determines the activation of proteins of the UPR pathway involved in the induction of apoptosis, such as eukaryotic translation initiation factor 2A (eIF2A), activating transcription factor 4 (ATF4), and C/EBP Homologous Protein (CHOP) [165,166,167]. A proposed mechanism through which SFAs induce ER stress is the inhibition of the sarco/endoplasmic reticulum/Ca^2+^/ATPase pump activity and the consequent depletion of ER calcium [166,168]. Boslem et al. [132] hypothesized that lipotoxicity alters sphingolipid metabolism by reducing sphingomyelin in the ER, disrupting ER lipid rafts, and thus promoting protein traffic disorders and ER stress onset. Of note, ER stress can, in turn, activate neutral sphingomyelinase to generate ceramide, further participating in the reduction of sphingomyelin levels and the toxic accumulation of ceramides [169].

ER stress also appears to play a critical role in lipotoxicity- and obesity-induced insulin resistance in the liver, adipose tissue, and muscle tissue. An increased expression of ER stress markers, such as immunoglobin heavy chain-binding protein (Bip), protein kinase like ER kinase (PERK), and eIF2a is associated with an alteration of insulin signaling in the liver and adipose tissue of HFD-fed mice [170]. ER stress also mediates skeletal muscle insulin resistance in obese and diabetic patients [171]. Overall, ER stress could represent a link between lipotoxicity and insulin resistance in T2D.

Similarly, AD is considered a “protein misfolding disease”, characterized by a progressive aggregation of misfolded and unfolded proteins that contribute to the onset of ER stress, leading to neuronal dysfunction and death [172,173]. Lipotoxicity can promote ER stress in the brain: exposure of neuronal cells to palmitate triggers ER stress through the increased expression of eIF2A and BiP [174,175]. Moreover, a diet rich in palmitate causes ER stress in the brain, with consequent activation of CHOP in C57bL/6 mice, in vivo [176].

Of importance, high levels of FAs in the brain can indirectly contribute to ER stress through the accumulation of Aβ. In fact, in vitro studies report that Aβ fibrils or oligomers trigger ER stress [177,178,179,180] through mechanisms involving reduced ER Ca^2+^, mitochondrial dysfunction, and subsequent ROS production [181,182]. ER stress can, in turn, contribute to the formation of Aβ by increasing β-secretase levels, thus suggesting that ER stress and amyloid deposits can influence each other, resulting in an exacerbated effect [183,184,185].

ER stress has also been implicated in developing brain insulin resistance in mice fed a HFD, thus representing a key mechanism underlying the cognitive dysfunction typical of diseases such as AD [186].

### 2.7. Ferroptosis

Ferroptosis is a type of programmed cell death that is morphologically and biochemically distinct from others (e.g., autophagy, apoptosis, and necroptosis). Morphologically, ferroptosis is mainly characterized by the shrinkage of mitochondria with increased membrane density and the reduction or disappearance of mitochondrial cristae [187]. Biochemically, it is characterized by iron-dependent lipid peroxide accumulation and inadequate redox enzymes levels, such as glutathione peroxidase 4 (Gpx4) that converts glutathione (GSH) to oxidized glutathione in order to control ROS production by lipid peroxidation [188].

Pancreatic beta-cells are prone to ferroptotic death since they are extremely susceptible to oxidative stress and the accumulation of toxic lipid peroxides due to the low presence of detoxifying enzymes, as mentioned above (see Section 2.3). Of note, FFAs can deplete GSH, thus debilitating the detoxification of lipid peroxides by Gpx4 in pancreatic beta-cells [189,190,191].

Lipotoxicity can also alter lipid metabolism, thus inducing lipid peroxides accumulation and ferroptosis. Interestingly, in vitro studies have demonstrated that mainly ω-6- polyunsaturated FAs (PUFAs) are responsible for lipid peroxidation and the subsequent ferroptosis induction, whereas SFAs are more frequently linked to apoptosis in beta-cells [190].

Additionally, lipotoxicity can dysregulate the Fe-uptake by cytosolic transporter aconitase in beta-cells, thus resulting in uncontrolled Fe-uptake also into the mitochondria. The resulting mitochondrial Fe-overload, associated with alterations of lipid metabolism and oxidative stress, promotes the generation of lipid peroxides, resulting in ferroptosis activation in beta-cells [189].

In an early stage, ferroptosis has also been shown to alter insulin biosynthesis and secretion in pancreatic beta-cells [192]. As a result of its deleterious effects on both beta-cell function and survival, this type of programmed cell death has been associated with the pathogenesis of diabetes.

As for pancreatic beta-cells, the scarcity of antioxidant defense mechanisms makes the human brain highly susceptible to ROS insults and oxidative stress, therefore, more prone to FFAs-induced ferroptosis [92,193]. Indeed, lipotoxicity reduces endogenous antioxidant systems, including Gpx4, also in the brain, and this is closely linked to ferroptosis and AD pathology [193,194].

### 2.8. Autophagy

Autophagy is a physiological process that occurs in cells to remove damaged and dysfunctional organelles and cytoplasmic material, which have the potential to be cytotoxic [151]. Although autophagy is maintained at low levels in cells to control homeostasis in biological processes, it can also be strongly activated to achieve a defensive cellular response to lipotoxicity and associated ER stress [195]. However, prolonged autophagy activation can cause significant damage to the intracellular environment and trigger a programmed cell death, also known as “autophagic cellular apoptosis” [195].

Interestingly, autophagy dysregulation has been linked to the onset and progression of both T2D and AD, since it can affect beta-cell function and survival, as well as mediate the aggregation of Aβ and tau deposits in the brain [196,197,198].

Of note, under lipotoxic conditions, the autophagic process is strongly triggered by ER stress in beta-cells, although the molecular mechanisms underlying this event have not been fully identified. It has been suggested that ER stress could promote autophagy by activating the JNK protein or by regulating the Akt/Tuberous Sclerosis Complex/mTOR pathway. In the latter case, the inhibition of Akt phosphorylation results in increased autophagy, impaired insulin signaling, and pancreatic beta-cell dysfunction [199].

In vivo studies have demonstrated the presence of increased autophagy in pancreatic beta-cells of both diabetic mice and nondiabetic HFD-fed mice. Of note, autophagy-deficient mutants mice fed with HFD showed profound deterioration of glucose tolerance, partly due to the lack of HFD-induced compensatory increase in beta-cell mass, suggesting a role of autophagy in guaranteeing the adaptive response of beta-cells to HFD-induced insulin resistance [200].

Autophagy is also involved in neuron and astrocyte physiology. Therefore, the impairment of any step of the autophagic pathway can generate axonal defects, culminating in neuronal degeneration and astrocyte damage [28]. It has been demonstrated that astrocytes can promote autophagy to prevent the formation of protein aggregates in neurons. However, as in beta-cells, persistent lipotoxic stimuli mediated by ceramide overaccumulation and ER stress may affect autophagy flux in the brain with the subsequent activation of apoptotic signaling [28]. In conclusion, basal autophagy represents a protective mechanism also in the brain, but lipotoxicity-induced autophagy dysregulation can induce cellular apoptosis or necrosis, brain damage, and dysfunction, thus suggesting a role in AD pathogenesis [201].

## 3. Possible Common Therapies

The discovery of the existence of a causative link between T2D and AD has generated a great interest in exploring whether anti-diabetes drugs could also be beneficial for AD treatment [202,203], such that numerous clinical trials and animal studies have been conducted for this purpose in the last few years. These aspects have been recently reviewed by Michailidis et al. [202], leading to the conclusion that the high variability in the experimental design of the studies and heterogeneity in the results make it difficult to come to robust assumptions. However, some anti-diabetes drugs exert some beneficial effects on working memory, as well as on cognitive and functional abilities, both on humans and mice. For instance, Logroscino et al. demonstrated that women with diabetes showed a worse cognitive status than women without diabetes. However, the use of oral hypoglycaemic agents fully recovered their cognitive performance [204]. Among the well-established therapeutic options for T2D treatment, insulin and glucagon-like peptide 1 receptor agonists (GLP-1RAs) have shown the most promising results in relieving typical AD traits.

Insulin exerts many important functions in the brain, mostly regulating food intake and energy homeostasis [205]. It can also reduce tau protein hyperphosphorylation, enhance Aβ clearance and synaptic plasticity, and improve memory [206,207,208]. In particular, intranasal insulin appeared to be an effective therapeutic approach for patients with AD, improving working memory and cognitive skills, with no side effects due to prolonged treatment [209,210]. Of note, intranasal insulin, unlike peripheral insulin administration, has the advantage of bypassing the BBB and avoiding the risk of hypoglycemia [211]. Only a few studies have suggested the ability of exogenous insulin to prevent lipotoxicity-induced injury of pancreatic beta-cells [212] and skeletal muscle cells [213]. Conversely, to the best of our knowledge, no studies have investigated the anti-lipotoxic effects of insulin in brain cells. However, insulin delivered to the brain by intranasal administration is known to acutely suppress systemic lipolysis and reduce circulating FFAs levels in humans, which could lead to the prevention of lipotoxicity-induced neurodegeneration [213].

GLP-1RAs (in particular, exenatide and liraglutide) have also shown interesting effects in neuroregulation and neuroprotection. Indeed, GLP-1R is widely distributed in the brain and specifically in the hypothalamus, thalamus, brain stem, striatum, substantia nigra, cerebral cortex, and hippocampus [214]. Notably, numerous GLP-1RAs have been successfully used in rescuing AD animal models [215], showing a marked ability to enhance neuronal cell proliferation, memory, and synaptic plasticity, while reducing Aβ accumulation, oxidative stress, and neuroinflammation [216]. Despite this, only a few trials have explored the effects of GLP-1RAs in human AD patients. Among them, Mullins et al. [217] showed that 18 months of treatment with exenatide on patients at risk of AD did not produce differences in several cognitive parameters, magnetic resonance imaging of cortical thickness and cortical volume compared to placebo. Additional two trials confirmed no cognitive differences in patients with AD or cognitive impairment treated with liraglutide [218,219]. Conversely, Gejl et al. [220] demonstrated that the six-month treatment of AD patients with the GLP-1RA liraglutide had moderate neuroprotective effects, mainly expressed by improvements in cerebral glucose metabolism, without effects on amyloid deposition or cognition. Interestingly, among the anti-diabetes drugs with potential beneficial effects in patients with AD, GLP-1RAs are those with the most pronounced anti-lipotoxic effects not only in peripheral organs (skeletal muscle, heart, liver, adipose tissue, and pancreas) [221,222], but also in the brain [223,224,225,226]. However, in the latter case, there are still few studies. In particular, GLP-1RAs are able to attenuate lipotoxicity-induced oxidative stress [227] and inflammation [228,229] in hepatic HepG2 cells and the liver of HFD-fed mice. Interestingly, Leonardini et al. [230] have demonstrated that the activation of GLP-1R counteracts palmitate-induced apoptosis via inhibition of ceramide generation in human cardiac progenitor cells. Furthermore, numerous studies have demonstrated the ability of GLP-1RAs to prevent lipotoxicity-induced beta-cell failure by targeting numerous dysfunctional pathways, such as inflammation, oxidative stress, ER stress, and, to a lesser extent, autophagy and amyloid accumulation (reviewed in [231]). Although ad hoc studies are needed both in vitro and in animal models, GLP-1RAs are very likely to exert their anti-lipotoxic effects on the brain as well.

In addition to drugs, lifestyle modification (increased physical activity and healthy dietary intervention) is one of the first management strategies advised for patients newly diagnosed with T2D and should accompany the diabetic patient throughout his life [232]. Similarly, since no effective pharmacological treatment is available to cure AD, a greater emphasis has been placed on implementing non-pharmacological (lifestyle) interventions that may prevent AD or reduce the escalation of AD burden [233]. Targeted studies will have to be conducted to identify how a physical activity or specific functional foods can positively impact the onset and progression of both T2D and AD, focusing on the mechanisms underlying lipotoxicity. However, some examples are already present in the literature.

For instance, irisin is a hormone mainly secreted by skeletal muscle in response to physical activity, with a pivotal role in regulating energy metabolism [221,234]. Numerous interventional studies in animal models of diabetes and/or obesity have shown that the exogenous administration of recombinant irisin can restore glucose and lipid homeostasis and exert anti-diabetic and anti-obesity effects [221]. Irisin has shown prominent anti-lipotoxic effects in the heart, skeletal muscle, liver, and pancreatic beta-cells [221,235]. In addition, recent evidence suggests that irisin plays a developmental role in regulating the process of neuronal differentiation and maturation, induces the expression of neurotrophic factors, such as brain-derived neurotrophic factor (BDNF), and could exert neuroprotective effects on neurodegenerative diseases, improving memory impairment and synaptic plasticity [236,237]. Interestingly, irisin improves learning and memory function, regulates cognitive function, promotes neurogenesis, and prevents neuronal damage caused by oxidative stress, thus representing a potential target for ameliorating AD pathology and preventing AD onset [238,239,240,241]. Of note, irisin levels are reduced in both the serum of T2D patients [242] and the cerebrospinal fluid of AD patients [239].

On the other hand, a healthy dietary pattern, characterized by antioxidant and anti-inflammatory properties, significantly reduces the risk of T2D, especially in the high-risk population [243]. At the same time, it may constitute promising approaches in preventing cognitive decline or delaying the progression to AD [233]. For instance, the adoption of a diet pattern particularly rich in PUFAs and polyphenols (e.g., curcumin, apigenin, resveratrol, quercetin, and many others) has long been recognized as a promising strategy to prevent the onset and progression of both T2D [243,244,245,246,247,248] and AD [249,250,251,252,253]. Their ability to prevent lipotoxic damage in the setting of T2D and AD remains largely to be demonstrated.

## 4. Conclusions

Over the past years, although numerous trials have been performed in AD patients, only two of them have led to the approval of a medication for their clinical use (memantine in 2003 and aducanumab in 2021), proving that drug development in AD is indeed challenging. The discovery of a causative correlation between T2D and AD has generated a great interest in exploring whether anti-diabetes pharmacological and non-pharmacological therapeutic approaches could also be beneficial for AD treatment. In this regard, the most promising approaches are represented by lifestyle modifications, intranasal insulin, and GLP-1RAs. Nevertheless, the scarcity of the studies, the high variability in these studies, and the heterogeneity in the results make it difficult to come to robust conclusions.

**Figure 1:**
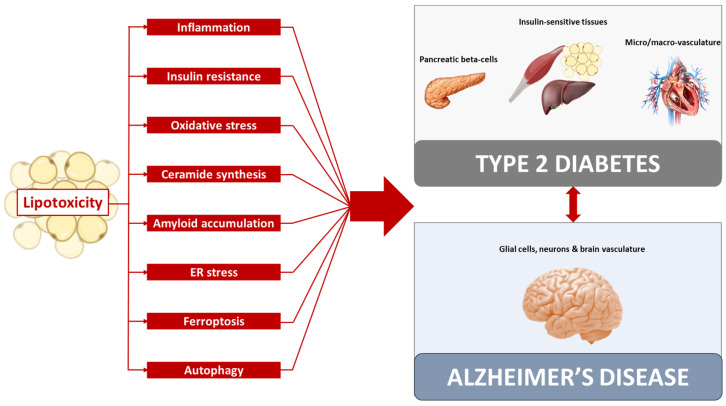
Lipotoxicity causes the activation of damage pathways, including inflammation, insulin resistance, oxidative stress, ceramide synthesis, amyloid accumulation, ER stress, ferroptosis, and autophagy. Through the activation of these pathways, lipotoxicity can promote the dysfunction of pancreatic beta-cells, insulin-sensitive tissues, and micro/macro-vasculature, thus favoring the onset and progression of type 2 diabetes, as well as the dysfunction of glial cells, neurons, and brain vasculature, thus favoring the onset of Alzheimer’s disease. Therefore, lipotoxicity may represent a common trigger in the pathogenesis of type 2 diabetes and Alzheimer’s disease, which could explain the strong correlation between these two pathological conditions.

In order to envision new molecules with therapeutic efficacy in both T2D and AD, it is important to know the molecular pathways involved in the onset and progression of both diseases. In this review, we have reported evidence that lipotoxicity may represent the common trigger in the pathogenesis of T2D and AD. Indeed, lipotoxicity can promote pancreatic beta-cell dysfunction and peripheral insulin resistance, which are the two hallmarks of T2D, as well as to promote neurodegeneration and neuronal dysfunction typical of AD. Interestingly, the lipotoxicity-induced damage pathways are very similar in all tissue involved in the pathogenesis of both T2D and AD and include inflammation, insulin resistance, oxidative stress, ceramide synthesis, amyloid accumulation, ER stress, ferroptosis, and autophagy (Figure 1). This evidence suggests that lipotoxicity represents a crucial biological feature that unites T2D and AD, and therefore it should be targeted in the design of new drugs with anti-diabetes and anti-neurodegeneration effects.

## Data Availability

Not applicable.
